# Induction of Pluripotent Stem Cells from a Manifesting Carrier of Duchenne Muscular Dystrophy and Characterization of Their X-Inactivation Status

**DOI:** 10.1155/2017/7906843

**Published:** 2017-04-12

**Authors:** Yuko Miyagoe-Suzuki, Takashi Nishiyama, Miho Nakamura, Asako Narita, Fusako Takemura, Satoru Masuda, Narihiro Minami, Kumiko Murayama, Hirofumi Komaki, Yu-ichi Goto, Shin'ichi Takeda

**Affiliations:** ^1^Department of Molecular Therapy, National Institute of Neuroscience, National Center of Neurology and Psychiatry, 4-1-1 Ogawahigashi, Kodaira, Tokyo 187-8502, Japan; ^2^Department of Laboratory Medicine, National Center Hospital for Mental, Nervous and Muscular Disorders, National Center of Neurology and Psychiatry, Kodaira, Tokyo 187-8502, Japan; ^3^Department of Child Neurology, National Center Hospital, National Center of Neurology and Psychiatry, Kodaira, Tokyo 187-8551, Japan; ^4^Department of Mental Retardation and Birth Defect Research, National Institute of Neuroscience, National Center of Neurology and Psychiatry, 4-1-1 Ogawahigashi, Kodaira, Tokyo 187-8502, Japan

## Abstract

Three to eight percent of female carriers of Duchenne muscular dystrophy (DMD) develop dystrophic symptoms ranging from mild muscle weakness to a rapidly progressive DMD-like muscular dystrophy due to skewed inactivation of X chromosomes during early development. Here, we generated human induced pluripotent stem cells (hiPSCs) from a manifesting female carrier using retroviral or Sendai viral (SeV) vectors and determined their X-inactivation status. Although manifesting carrier-derived iPS cells showed normal expression of human embryonic stem cell markers and formed well-differentiated teratomas in vivo, many hiPS clones showed bi-allelic expression of the androgen receptor (AR) gene and loss of X-inactivation-specific transcript and trimethyl-histone H3 (Lys27) signals on X chromosomes, suggesting that both X chromosomes of the hiPS cells are in an active state. Importantly, normal dystrophin was expressed in multinucleated myotubes differentiated from a manifesting carrier of DMD-hiPS cells with XaXa pattern. AR transcripts were also equally transcribed from both alleles in induced myotubes. Our results indicated that the inactivated X chromosome in the patient's fibroblasts was activated during reprogramming, and XCI occurred randomly during differentiation.

## 1. Introduction

X-linked Duchenne muscular dystrophy (DMD) is caused by mutations in the *DMD* gene, which encodes the dystrophin protein required for stability of the sarcolemma. Most female carriers of *DMD* mutations are asymptomatic, but 3–8% of female carriers develop symptoms ranging from a DMD-like progression to a very mild Becker muscular dystrophy-like phenotype [1] due to skewed inactivation of X chromosomes in early development [2–4].

Human induced pluripotent (iPS) cells are embryonic stem- (ES-) like pluripotent cells derived from somatic cells by ectopic expression of a defined set of reprogramming factors [5, 6]. Patient-derived iPS cells are expected to be useful for disease modeling, but the effects of reprogramming by Yamanaka factors on X-inactivation in female iPS cells remain controversial. A previous study showed that human iPS cells exhibit a nonrandom X chromosome inactivation (XCI) pattern because they reflect the XCI status of the single fibroblast from which they were derived [7]. Other groups reported two active X chromosomes in iPS cells derived from a patient with Rett syndrome [8]. For disease modeling of a manifesting carrier of DMD in vitro, the correct understanding of the XCI status of female iPS cells and hiPS-derived skeletal muscle is needed.

Here, we established iPS cells from one female DMD-manifesting carrier and one female DMD carrier with three X chromosomes and high serum creatine kinase (CK) levels by using an all-in-one retroviral vector or Sendai viral (SeV) vector and examined their X-inactivation status. Many hiPS clones showed a loss of X-inactivation-specific transcript (XIST) RNA and loss of biased methylation in exon 1 and bi-allelic expression of the *androgen receptor* (AR) gene. Interestingly, skeletal muscle cells differentiated from manifesting carrier of DMD-derived hiPSCs with XaXa patterns expressed dystrophin. Our results suggest that the inactivated X chromosome in the female manifesting carrier of DMD was activated during reprogramming, and XCI occurred randomly on differentiation.

## 2. Materials and Methods

### 2.1. Patient Fibroblasts

Patient 609 (41 years old) is a manifesting carrier of Duchenne muscular dystrophy. Dystrophin staining of muscle sections showed a mosaic pattern. Western blotting showed that the dystrophin protein level was 10% of the normal. Multiplex PCR revealed deletion of dystrophin exons 42-43 of the DMD gene (frame-shift mutation). Patient 386 is a 5-year-old girl with XXX trisomy. MLPA analysis revealed deletion of exons 13–44 in one X. The patient shows high levels of serum CK but no obvious muscle weakness. Patient 401 (1y2m, male) has duplication of exons 45–50 of the DMD gene. The generation and analysis of iPS cell lines and deposition of these cell lines in a public cell bank (RIKEN Cell Bank) were approved by the patients or their parents using consent forms and approved by NCNP Ethics Committees. Samples were anonymized upon leaving the clinic.

### 2.2. Reprogramming by Yamanaka Factors

#### 2.2.1. Retroviral Vectors

**Figure 1:**
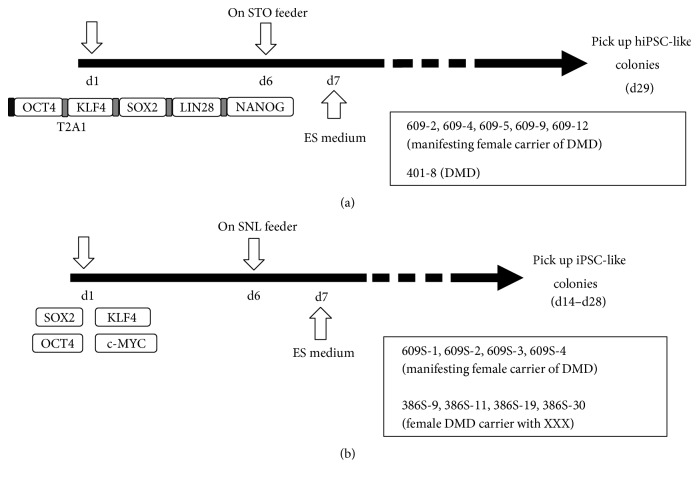
Time schedules of hiPS induction using an all-in-one retroviral vector (a) or four Sendai viral vectors (b). Fibroblasts from a manifesting female carrier of DMD (609) were infected with a retroviral vector, encoding five reprogramming factors. After replating onto MMC-treated STO feeder cells, colonies were picked up (a). Next, fibroblasts of 609 and a female DMD patient (386) were infected with four Sendai viral vectors on SNL feeders, and ES-like colonies were picked up 14–21 days after transduction (b).

Fibroblasts from patient 609 were infected with the human iPS cell generation all-in-one retroviral vector pDON-5 OKSNL (Takara Bio, Japan), encoding all five reprogramming factors (OCT4, KLF4, SOX2, LIN28, and NANOG), and then replated on STO cells. Virus particles were prepared using a retrovirus packaging kit Amph0 (Takara Bio) and a G3T-hi packaging cell line (Takara Bio). Human ES cell-like colonies were picked up at day 29 (Figure 1). Reprogramming efficiency (ALP + colonies/starting cell numbers) was 0.19%. Finally, five clones were selected based on their morphology and growth rates. STR analysis was performed to confirm that these iPS clones were derived from patient 609's fibroblasts. 401-8 iPS cells were obtained from 401 fibroblasts using the same protocol with 609 iPS clones.

#### 2.2.2. Sendai Viral Vectors

To reprogram fibroblasts of patients 609 and 386, we used CytoTuneTM-iPS (DNAVEC, Tsukuba, Japan). Vector cocktails (*OCT3/4-SeV/TSΔF*, *SOX2-SeV/TSΔF*, *KLF4-SeV/TSΔF*, and *c-MYC(HNL)-SeV/TS15ΔF*) were used at an MOI of 3, according to the manufacturer's protocol. Human ES cell-like colonies were grown on SNL feeder cells, and picked up on days 14–28. RT-PCR for transgenes and the SeV genome were performed using the following primers: for SeV, forward: GGA TCA CTA GGT GAT ATC GAG C and reverse: ACC AGA CAA GAG TTT AAG AGA TAT GTA TC (181 bp); for c-MYC, forward: TAA CTG ACT AGC AGG CTT GTC G and reverse: TCC ACA TAC AGT CCT GGA TGA TGA TG (532 bp); for KLF4, forward: ACA AGA GAA AAA ACA TGT ATG G and reverse: CGC GCT GGC AGG GCC GCT GCT CGA C (529 bp); for SOX2, forward: ACA AGA GAA AAA ACA TGT ATG G and reverse: ATG CGC TGG TTC ACG CCC GCG CCC AGG (591 bp); for OCT3/4, forward: CCC GAA AGA GAA AGC GAA CCA G and reverse: AAT GTA TCG AAG GTG CTC AA (483 bp) (Integrated DNA Technologies, Inc.). Sendai iPS cells were immunostained with anti-Sendai virus polyclonal antibodies (MBL).

### 2.3. hiPS Cell Culture

hiPS cells were maintained on mitomycin C- (MMC-) treated SNL feeder cells (CiRA), MMC-treated mouse embryonic fibroblasts (MEFs), or SL10 feeder cells (ReproCELL) in a primate ES cell medium (ReproCELL) supplemented with bFGF (4 ng/ml). Human iPS cells, 201B7 [5], 253G1 [9], and 409B2 [10], were provided by S. Yamanaka of Kyoto University and used as control iPS cell lines.

### 2.4. Immunocytochemistry

Cells were fixed with 4% paraformaldehyde for 5 min, permeabilized with 0.1% Triton-X in PBS for 10 min, blocked with 5% goat (Cedarlane) or horse (Invitrogen) serum in 2% BSA for 15 min, and then incubated with anti-TRA-1-60 mouse monoclonal antibody (Millipore), anti-TRA-1-81 mouse monoclonal antibody (Millipore), anti-SSEA3 rat monoclonal antibody (R&D), anti-NANOG polyclonal antibody (R&D), anti-SOX2 antibody (6F1.2) (Millipore), anti-OCT4 mouse monoclonal antibody (C-10) (Santa Cruz Biotechnology), or anti-trimethyl-histone H3 (Lys27) (polyclonal, Millipore). The cells were then incubated with secondary antibodies labeled with Alexa-Fluor 488 or 568 (Molecular Probes) and DAPI. Images were photographed using a fluorescence microscope IX71 (Olympus, Tokyo, Japan) equipped with an Orca2 air-cooled CCD camera (Hamamatsu Photonics) and AQUACOSMOS software (Hamamatsu Photonics).

### 2.5. Fluorescence-Activated Cell Sorting (FACS)

Cells were resuspended at a concentration of 1.0 × 10^6^ cells/100 *μ*l in PBS containing 2% FBS and incubated with anti-SSEA4 (MC-813-70, Millipore), anti-TRA-1-60 (Millipore), or anti-TRA-1-81 (Millipore) antibodies on ice for 30 min, then incubated with Alexa 488-labeled goat anti-mouse IgM (Invitrogen) or Alexa 488-labeled rabbit anti-mouse IgG (Invitrogen). After extensive washing, cells were analyzed on an FACSAria flow cytometer (BD Bioscience).

### 2.6. RT-PCR and RT-PCR Array

Total RNA was extracted from cells using a MicroRNeasy kit (Qiagen). Complementary DNA (cDNA) was synthesized using a QuantiTect reverse transcription kit (Qiagen). For expression of DMD, four PCR primers were used: ex42F, AAT CAC TCA TGT CTC ACA AGC CCT A; ex43R, TCC GAC CTG AGC TTT GTT GTA; ex44F, TCC TGA GAA TTG GGA ACA TGC TA; and ex45R, CCA GTT GCA TTC AAT GTT CTG AC. For expression of AR, AR-F (TCCAGAATCTGTTCCAGAGCG TGC) and AR-R (CTCTACGATGGGCTTGGGGAGAAC) were used. For RT-PCR array, the gene expression was analyzed using a human embryonic stem cell RT2 profiler (SABiosciences, Qiagen) PCR array according to the manufacturer's protocol.

### 2.7. Karyotype G-Band Staining

Analysis was performed by Nihon Gene Research Laboratories Inc. (Sendai, Japan). The numbers of chromosomes were checked in fifty cells per group, then 20 cells were further analyzed by detailed investigation.

### 2.8. Androgen Receptor Exon 1 Methylation Analysis

Genomic DNA extracted from hiPS cells and parental fibroblasts were digested with HpaII or MspI. Then, digested DNA was amplified with Ar1-r-6FAM primer (6FAM TCCAGAATCTGTTCCAGAGCG TGC) and Ar1-f2 primer (CTCTACGATGGGCTTGGGGAGAAC). The PCR products were analyzed on a 3130xl Genetic Analyzer (Applied Biosystems), and the ratios of signals were quantitated as described [11]. Male genomic DNA was used as a control for complete digestion of the unmethylated X chromosome (no signal). Since the difference between the two bands (a and b) was only 3 bp (23 CAG repeats and 24 CAG repeats, resp.) in patient 609, we detected the overlap of stutter bands. To revise the signal intensity, we used the following formula: true  *b* = *b* – *a* × 0.31. The calculation of 0.31 was based on our observation on 24 samples of 8 individuals.

### 2.9. Direct Sequencing of Androgen Receptor Transcripts

RNAs isolated from iPS cells were reverse-transcribed into cDNA and amplified with Ar1-r-6FAM and Ar1-f2 primers. Sequences of the PCR products were determined by direct sequencing. PCR products from induced myotubes were then cloned into a TA cloning vector (Invitrogen) and inserts were sequenced.

### 2.10. Fluorescence In Situ Hybridization (FISH) with XIST RNA Probe and X Chromosome Probe

FISH analysis was performed on parental fibroblasts and hiPS cells at Chromosome Science Labo Inc., Sapporo, Japan (http://www.chromoscience.jp). In brief, exon 1 (6 kb) and exon 6 (6 kb) of the XIST gene were amplified by PCR and labeled with Cy3 by nick translation. For the X chromosome, an FITC-labeled HXO-10 probe (Chromosome Science Labo Inc.) that hybridizes with the DXZ1 region was used. Cells on chamber slides were treated with 0.01% pepsin/0.1 N HCl for 4 min and then fixed in 4% paraformaldehyde/PBS. After denaturing at 70 °C for 5 min, cells were incubated with an XIST probe and X probe. After washing with 50% formamide/2xSSC, and 1xSSC, cells were stained with DAPI and mounted. The images were taken using a Leica DMRA2 camera and Leica CW4000 FISH software.

### 2.11. Teratoma Formation Assay

hiPSCs (1 × 10^6^) were injected into the testes of 8- to 12-wk-old *NOD/Scid* mice. Three months after injection, tumors were dissected and fixed in 15% formalin, embedded in paraffin, cut by a microtome, and stained with hematoxylin and eosin (H&E).

### 2.12. Myogenic Induction by Inducible MyoD

hiPS cells were cultured on iMatrix-511 (Nippi) in StemFit AK02N culture medium (Ajinomoto) and induced into the mesoderm lineage using STEMDiff Mesoderm induction medium (Stem Cell Technologies). After 3 d culture, the cells were cultured as spheres [12]. After 6 wk culture, the cells were plated onto collagen-coated plates and infected with a lentiviral vector encoding a doxycycline (dox)-inducible mouse MyoD gene (pLVi(3G)-TagGFP-E2a-MYOD-Puro, Sirion Biotech). After 2 d selection with puromycin (1 *μ*g/ml) (Calbiochem) and dox (1 *μ*g/ml) (LKT Laboratories) selection, the cells were induced to form myotubes in the presence of dox in DMEM supplemented with 2% horse serum. Multinucleated myotubes were immunostained with Hoechst 33258 dye (nuclei), antidystrophin polyclonal antibody (Abcam), and antimyosin heavy chain (MF20) (R&D Systems), and then visualized with goat anti-mouse IgG2b-Alexa568 and goat anti-rabbit IgG-Alexa 488 (Life Technologies).

## 3. Results

### 3.1. Induction of iPS Cells from Fibroblasts of a Manifesting DMD Carrier

**Figure 2:**
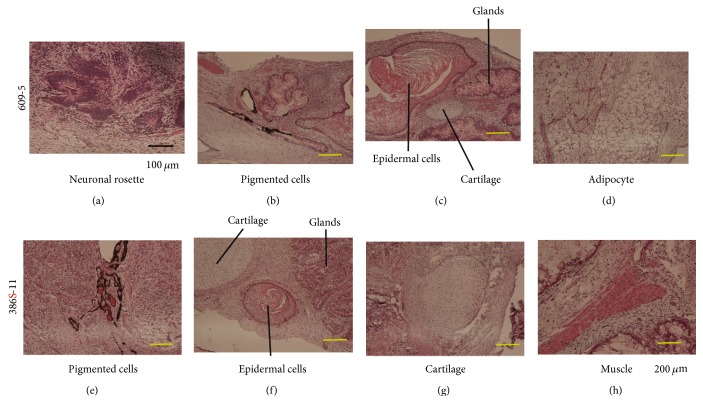
In vivo differentiation of 609-5 iPS cells (XX) and 386S-11 (XXX) iPS cells. Six months after transplantation into the testes of NOD/Scid mice, hiPS cells (609-5: (a–d), 386S-11: (e–h)) had formed teratomas, including mesodermal tissues (adipocytes, cartilage, and muscle), endodermal tissues (mucus-producing epithelium), and ectodermal tissues (neuronal rosette, epidermal cells, and pigmented cells).

Fibroblasts from a manifesting carrier (patient 609) were first infected with an all-in-one retroviral vector expressing OCT4, KLF4, SOX2, LIN28, and NANOG and replated on STO feeder cells; five clones were obtained (609-2, 609-4, 609-5, 609-9, and 609-12), Next, the same fibroblasts were infected with four Sendai viral vectors encoding for SOX2, KLF4, OCT4, or c-MYC and replated on SNL feeder cells (Figure 1). We obtained four clones (609S-1, 609S-2, 609S-3, and 609S-4) using the Sendai viral vector. The appearance of ES-like colonies was faster (2-3 weeks) with SeV-mediated reprogramming than with retroviral vector-mediated reprogramming (3-4 weeks). SeV RNA (RT-PCR) or proteins were not detected as early as passage three (Supplementary Figure 2 available online at https://doi.org/10.1155/2017/7906843). The expression of human ES markers (TRA-1-60, TRA-1-81, SSEA3, NANOG, OCT3/4, and SOX2) in all hiPS cells was confirmed by immunocytochemistry (Supplementary Figure 2). FACS analysis confirmed the expressions of SSEA4, TRA-1-60, and TRA-1-81 on all hiPS cells tested (Supplementary Figure 3). Gene expressions of human ES markers and differentiation markers were also examined by RT-PCR array (Supplementary Figure 3). Patient 609's iPSCs showed similar gene expression patterns to 201B7 and 253G4, which have already shown pluripotency in both in vitro differentiation assay and teratoma formation experiments [5, 9] (Supplementary Figure 3). FISH analysis using an X chromosome probe and G-band staining of parental fibroblasts and of hiPS cells (609-4 iPS and 609S-3 iPS cells) revealed that parental fibroblasts and iPS cells have normal karyotypes (46XX) and no gross chromosomal abnormalities (Supplementary Table 1 and Supplementary Figure 1). hiPS cells derived from 609 fibroblasts formed well-differentiated teratomas three months after transplantation into the testis (Figure 2).

### 3.2. Methylation of Exon 1 of *AR* Gene and Allelic Expression of *AR* Gene in Parental Fibroblasts and hiPS Cells

**Figure 3:**
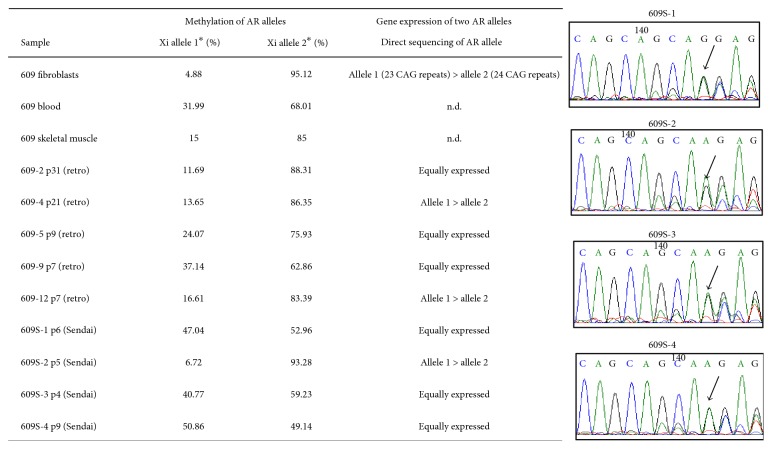
Methylation status and allele-specific expression of androgen receptor (AR) gene in the fibroblasts, skeletal muscle, and blood sample of a manifesting carrier of DMD (609) and derived iPS cells. *Left panel*: ^∗^% is calculated based on the assumption that one of two alleles is methylated, and signal intensities from allele 1 + allele 2 are shown as 100%. However, not all iPS clones have XCI. *Right panel*: representative images of direct sequencing of the *AR* gene.

**Figure 4:**
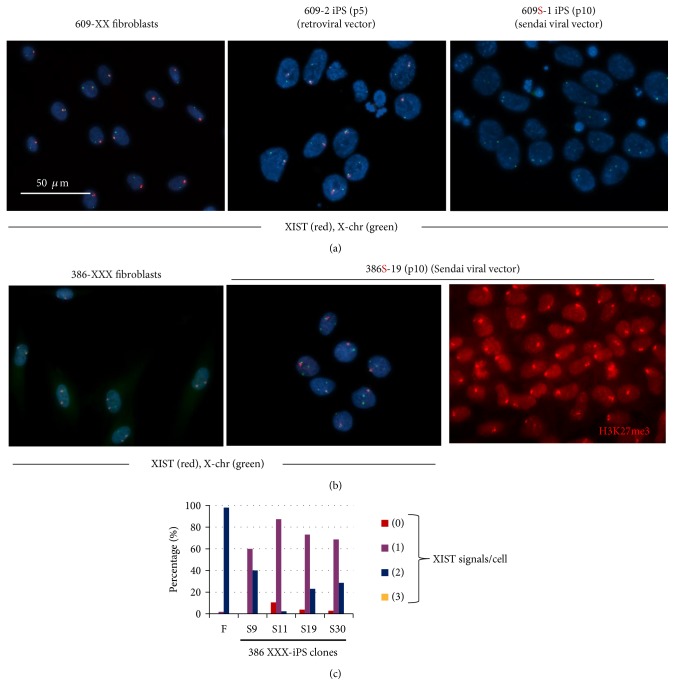
Loss of XIST signals in 609-iPS cells (XX) and 386-iPSCs (XXX). (a) Representative images of XIST RNA FISH (red)/X chromosome-DNA FISH (green) on 609 fibroblasts, 609-2 iPS cells (passage 5: p5), and 609S-1 iPS cells (p10). 609 fibroblasts have one XIST signal. Most 609-2 iPS cells retain XIST expression. In contrast, 609S-1 iPS cells, which were generated with Sendai viral vectors, had totally lost XIST expression. Quantitative data are also shown in Table 2. (b) Representative images of XIST RNA FISH (red)/X chromosome-DNA FISH (green) on 386 fibroblasts, and 386-S19 iPS cells. 386 fibroblasts have three X and two XIST signals. 386-S19 iPS cells, which were induced using Sendai viral vectors retained one XIST expression. Trimethyl-histone H3 (Lys27) (H3K27me3) signals (red) in 386-S19 iPS cells are shown. Most cells have just one H3K27me3 signal/cell. (c) XIST signals per cell in 386 fibroblasts and four 386 iPS cells. 386 fibroblasts have three X chromosomes and two XIST signals. In four 386 iPS clones, which were induced with Sendai viral vectors, most cells have one XIST signals.

To determine the X chromosome-inactivation status of iPS cells, we first examined the methylation of exon 1 of the androgen receptor in muscle fibroblasts, skeletal muscle (biopsied sample), and blood according to the methods described by Allen et al. [13] (Figure 3). We digested genome DNA with HpaII (sensitive to methylation of CpG) or MspI (digests methylated CpG), and then amplified the exon 1 regions by PCR with specific primers. This assay is widely used for measuring XCI skewing of DMD homozygous carriers [1]. Patient 609 showed 22 and 23 repeats of CAG in the first exon of the androgen receptor gene (data not shown). Quantification of the signals of methylated and unmethylated alleles indicated skewing of XCI toward the same X chromosome in the skeletal muscle and muscle fibroblasts. The blood sample showed no obvious skewing. The data indicate that the AR gene with 23 CAG repeats and the mutated DMD gene are on the same X chromosome, and this allele (allele 1) is dominantly active in skeletal muscle. We next examined the methylation status of exon 1 of the androgen receptor in patient 609's iPS cells (Figures 3 and 4). All five retro-iPS clones inherited the nonrandom methylation pattern of *AR* exon 1 of the parental fibroblasts; allele 2 was preferentially methylated, although a theoretical pattern, 0% : 100%, was not observed. Surprisingly, three out of four SeV-iPS clones showed an almost nonskewing pattern, suggesting loss of XCI.

To know which allele of two AR genes is transcribed in iPS clones, the androgen receptor transcripts were reverse-transcribed into cDNA, amplified, and directly sequenced. Surprisingly, 6 iPS clones out of 9 iPS clones showed equally expressed patterns. Only three iPS clones (609-4, 609-12, and 609S-2) retained preferential expression of allele 1 (Figure 3 and Supplementary Figure 5).

### 3.3. XIST RNA Signals in Fibroblasts of Manifesting Carrier of DMD and Derived iPS Cells

Next, we examined XIST expression in fibroblasts, retro-iPS cells, and SeV-iPS cells. *XIST* is a nonprotein coding RNA that localizes to the inactive human X chromosome [14, 15]. RNA-FISH analysis revealed that more than 80% of retro-iPS cells in clones 609-2 and 609-4 retained XIST expression in one of the two X chromosomes (Figure 4 and Table 1). In contrast, no XIST signals were detected in 609S-1 iPS cells or 609S-2 iPSCs (Figure 4 and Table 1). Because 609S-2 iPS cells showed skewed methylation of the AR exon 1 and dominant expression of one AR gene (Figures 3 and 4), we speculate that most 609S-2 iPS cells had lost XIST RNA but remained hypermethylated and transcriptionally suppressed. In contrast, our observations suggest that 609S-1 iPS cells reactivated the inactive X chromosome.

### 3.4. XCI of iPS Cells Derived from a Female Carrier of DMD with XXX Trisomy

To further examine the effects of reprogramming on XCI, we generated iPSCs from a female carrier with XXX trisomy (patient 386) by Sendai viral vectors. This patient has a large deletion of Ex13–Ex44 in the active X chromosome and high serum CK levels but shows no obvious muscle weakness due to an in-frame mutation. In this case, immunostaining of dystrophin indicated that the other two X chromosomes are completely inactivated (data not shown). Induced iPS clones showed ES cell-like gene expression patterns (Supplementary Figure 4) and triple XXX karyotypes (Supplementary Figure 1). XIST FISH analysis also showed that parental fibroblasts have three X chromosomes and that two of them are coated with XIST RNA. In contrast, the majority of patient 386's iPS cells showed only one XIST signal (Figure 4(b)). H3K27me3 is a repressive chromatin mark on inactive X chromosomes. Signals of H3K27me3 were also found on only one X chromosome in most 386 iPS cells (Figure 4(b)). In spite of such an aberrant XCI status, 386-iPSCs programmed by SeV vectors formed well-differentiated teratomas in the testes of NOD/Scid mice (Figure 2).

### 3.5. Dystrophin Expression in DMD-Manifesting Carrier hiPSC-Derived Myotubes

**Figure 5:**
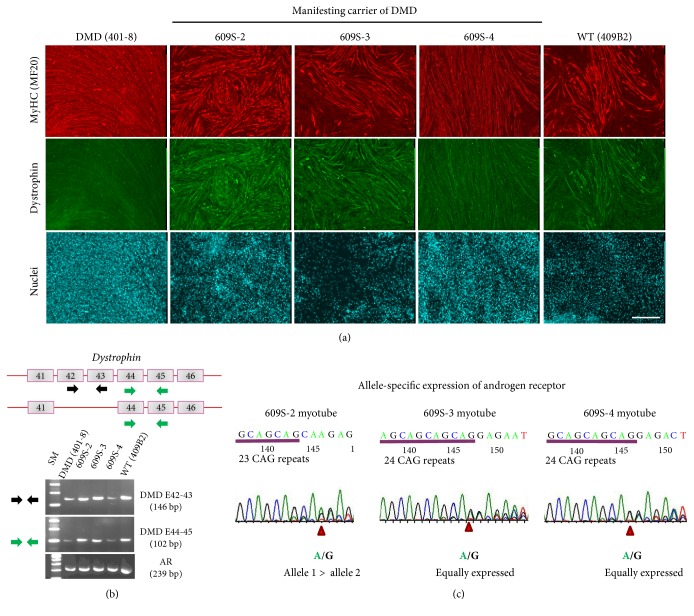
Myotubes derived from XaXa 609-hiPS cells restore normal dystrophin expression and nonskewed expression of the androgen receptor gene. (a) 409B2 iPSCs (normal), DMD-iPSCs (401-8, duplication of exons 45–50 of the dystrophin (*DMD*) gene), and manifesting carrier of DMD-iPS clones (609S-2, 609S-3, and 609S-4) were induced to differentiate into multinucleated myotubes. Normal iPS-derived myotubes expressed dystrophin. DMD-iPS-derived myotubes were negative for dystrophin. 609 iPSC-derived myotubes expressed dystrophin at comparable levels with wild-type myotubes. Scale bar: 500 *μ*m. (b) RT-PCR analysis for *dystrophin* (*DMD*) and *androgen receptor* (*AR*) in myotubes derived from 409B2 iPSCs (normal), DMD-iPSCs (401-8), and manifesting carrier of DMD-iPSCs (609S-2, 609S-3, and 609S-4). Primer positions and directions for dystrophin transcripts are indicated by arrows. (c) Direct sequencing of CAG repeats of AR transcripts in myotubes derived from manifesting carrier of DMD-iPS clones (609S-2, 609S-3, and 609S-4). 609S-2 myotubes show allele 1(23 CAG repeats)—dominant expression. In 609S-3 and 609S-4 myotubes, two alleles (23 and 24 CAG repeats) are equally expressed.

To determine whether patient 609's iPS cells indeed reactivated the X chromosome on which the normal dystrophin was encoded, we induced her iPS cells to differentiate into skeletal muscle lineage by using doxycycline-inducible MyoD expression and examined dystrophin expression by immunocytochemistry. Myotubes derived from 609S-2, 609S-3, and 609S-4 iPSCs expressed normal dystrophin at undistinguishable levels from myotubes derived from control 409B2 (Figure 5). RT-PCR for *dystrophin* confirmed that the normal *dystrophin* gene was actively transcribed. Direct sequencing of CAG-repeats of AR transcripts also indicated that both alleles are equally expressed in myotubes induced from 609S-3 and 609S-4 iPSCs. In 609S-2 myotubes, the AR gene with 23 CAG repeats on allele 1 was still preferentially expressed (Figure 5).

## 4. Discussion

### 4.1. Reactivation of Inactivated X Chromosome in hiPS Cells Is a Common Phenomenon

Inactivation of the X chromosome in human iPS cells has been well examined in normal female iPSCs [16] and Rett syndrome iPS cells [8, 17–20]. Three groups reported nonrandom XCI status of Rett iPS clones. In contrast, Marchetto et al. and Kim et al. reported that XCI was erased in some, but not all, reprogrammed RTT-iPSC clones, and again XCI occurred randomly during differentiation into neurons. The XCI of patient 609's iPS clones are similar to their RTT-iPSCs clones. We also found that XXX-iPS clones reprogrammed by SeV vectors have only one XIST signal, while the parental fibroblasts have two, suggesting activation of one of two inactive X chromosomes. Barakat et al. reported that many XXX-iPS cells derived from a triple X patient did not show Xi markers in their experiments [21]. Therefore, although we speculated that hiPS cells with three active X chromosomes cannot survive in an overdose of X chromosome genes, XaXaXa hiPS cells might survive in different culture conditions. To explain the contradictory results, further investigation would be required.

### 4.2. Do Reprogramming Methods Affect XCI of Female DMD-iPSCs?

We found a difference in XCI status between retro-iPSCs and Sendai-iPSCs, but the mechanisms by which different reprogramming methods produce hiPS cells with different XCI statuses is unclear. One possibility is that high levels and long duration of the expression of reprogramming factors by Sendai viral vectors cause a loss of XCI in hiPS cells. Tomoda et al. reported that the “Kyoto method,” in which LIF-producing SNL cells are used as feeders, contributes to reactivation of Xi during the reprogramming process [22]. Although how LIF erases XCI remains to be determined, our results are in agreement with their hypothesis; we used STO cells (parental cells of SNL cells) for retroviral reprograming, and SNL cells for Sendai viral-mediated reprogramming. We also compared the effects of two feeders, that is, MEFs and SL10, on reprogramming of normal female fibroblasts. Interestingly, reprogramming by MEFs tended to retain trimethyl-histone H3 (Lys27) compared with iPSCs established on SL10, again suggesting that the feeder cell is one factor that affects the XCI state (Supplementary Figure 6). Combination of reprogramming factors is another possible factor which influenced XCI in hiPSCs in our study (Figure 1). How both X chromosomes are activated during reprogramming and XaXa status was stably kept in culture should be determined in future studies.

### 4.3. Human iPSC Derived from a Manifesting Carrier of DMD Differentiated into Myotubes and Expressed Normal Dystrophin

Because many iPSC clones derived from a manifesting carrier of DMD showed XaXa-like patterns in methylation of the AR gene locus, expression of two *AR* alleles (Figure 3), XIST signals (Table 1, Figure 4), and H3K27me3 immunostaining (Figure 4), we expected the inactive X chromosome to be reactivated during reprogramming, resulting in a reset of skewed XCI in differentiated cells. Unexpectedly, “XaXa” hiPSCs derived from a DMD-manifesting carrier differentiated into multinucleated myotubes, and showed robust expression of dystrophin like the control. Our observation suggests that XaXa hiPSC clones reset the skewing of XCI to express dystrophin in differentiated cells. Bi-allelic *AR* expression in myotubes confirmed that XCI was randomly induced during differentiation from XaXa hiPSCs to myotubes (Supplementary Figure 7).

## 5. Conclusion

1. Many iPS clones derived from a manifesting female carrier of DMD had two active X chromosomes (XaXa) or mixed patterns (XaXa/XaXi).
2. Redifferentiation of female manifesting carrier of DMD-iPSCs with two active X chromosomes into myotubes restored dystrophin expression due to acquirement of nonskewed XCI.

## Supplementary Material

Supplementary TABLE 1 X-chromosome signals in the nucleus of fibroblasts isolated from a DMD-manifesting carrier (609) and in four hiPS clones.

## Figures and Tables

**Table 1:** XIST signals on the X chromosomes in the nuclei of fibroblasts isolated from a DMD-manifesting carrier (609) and four hiPS cells induced from 609 fibroblasts (609-2, 609-4, 609S-1, and 609S-2).

Cell (counted cells)	XIST (+) cells	XIST (−) cells
609 fibroblasts (*n* = 168)	164 (97.6%)	4 (2.4%)
609-2 p5 iPSCs (*n* = 151)	114 (75.5%)	37 (24.5%)
609-4 p6 iPSCs (*n* = 72)	62 (86.1%)	10 (13.9%)
609S-1 p10 iPSCs (*n* = 150)	0 (0%)	150 (100%)
609S-2 p8 iPSCs (*n* = 142)	0 (0%)	142 (100%)

N: number of cells counted; p: passage number.
