# Supplementary material for: Induction of Pluripotent Stem Cells from a Manifesting Carrier of Duchenne Muscular Dystrophy and Characterization of Their X-Inactivation Status
Source: Stem Cells Int. 2017 Apr 12;2017:7906843. doi: 10.1155/2017/7906843 (PMC5405591; doi:10.1155/2017/7906843)

Supplementary TABLE 1

X-chromosome signals in the nucleus of fibroblasts isolated from a DMD-manifesting carrier (609) and in four hiPS clones

X-Chromosome signals/nucleus	0	1	2	3	4
609 fibroblasts n=168	0	3 (1.8%)	155 (92.3%)	3 (1.8%)	7 (4.2%)
609-2 iPS (p5) (retroviral vector) n=151	0	2 (1.3%)	143 (94.7%)	2 (1.3%)	4 (2.6%)
609-4 iPS (p6) (retroviral vector) n=75	0	3 (4.0%)	69 (92.0%)	0	3 (4.0%)
609S-1 iPS (p10) (Sendai viral vector) n=150	1 (0.67%)	6 (4.0%)	137 (91.3%)	6 (4.0%)	0
609S-2 iPS (p8) (Sendai viral vector) n=142	0	4 (2.8%)	132 (93.0%)	4 (2.8%)	2 (1.4%)

Supplementary Figure 1

609 Fibroblast (parental)

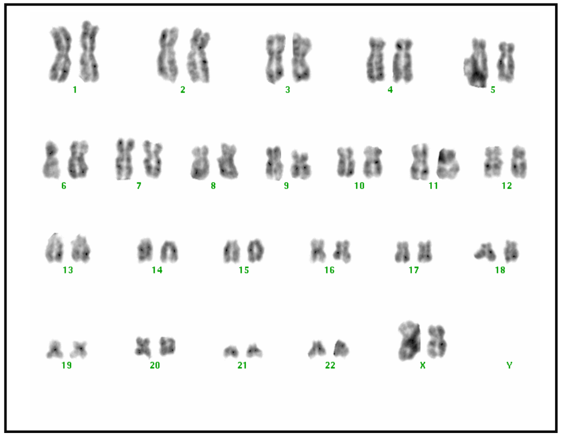

46, XX

609-4 (Retro-hiPSC, p6)

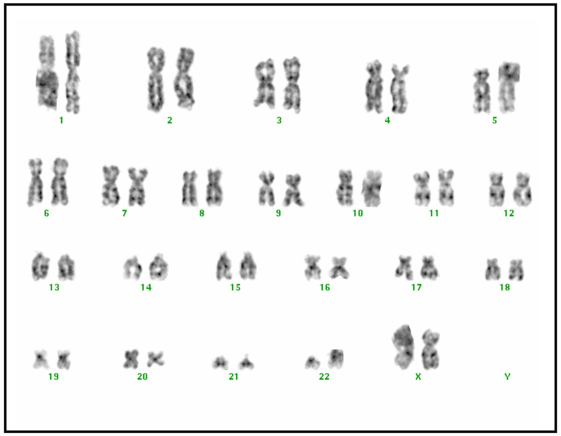

46, XX

609S-3 (Sendai-hiPSC, p5)

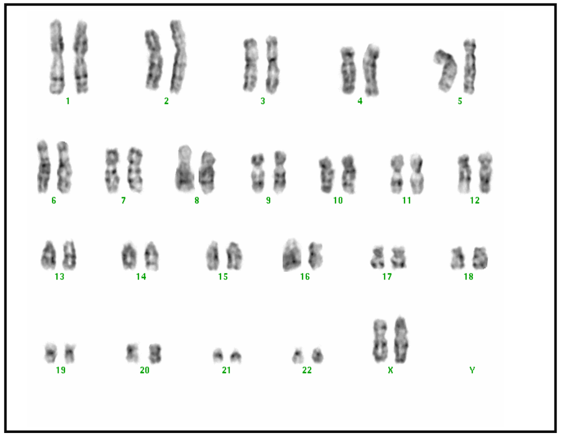

46, XX

386 Fibroblast (parental)

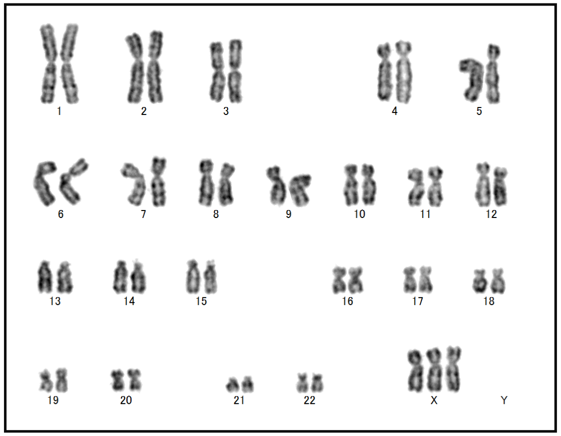

47, XXX

386S-11 (Sendai-hiPSC, p6)

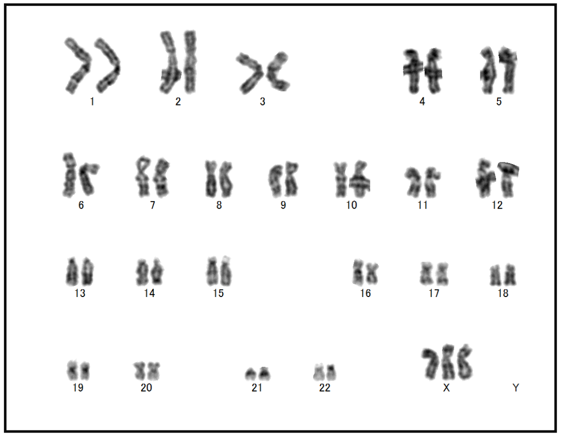

47, XXX

Supplementary Figure 2

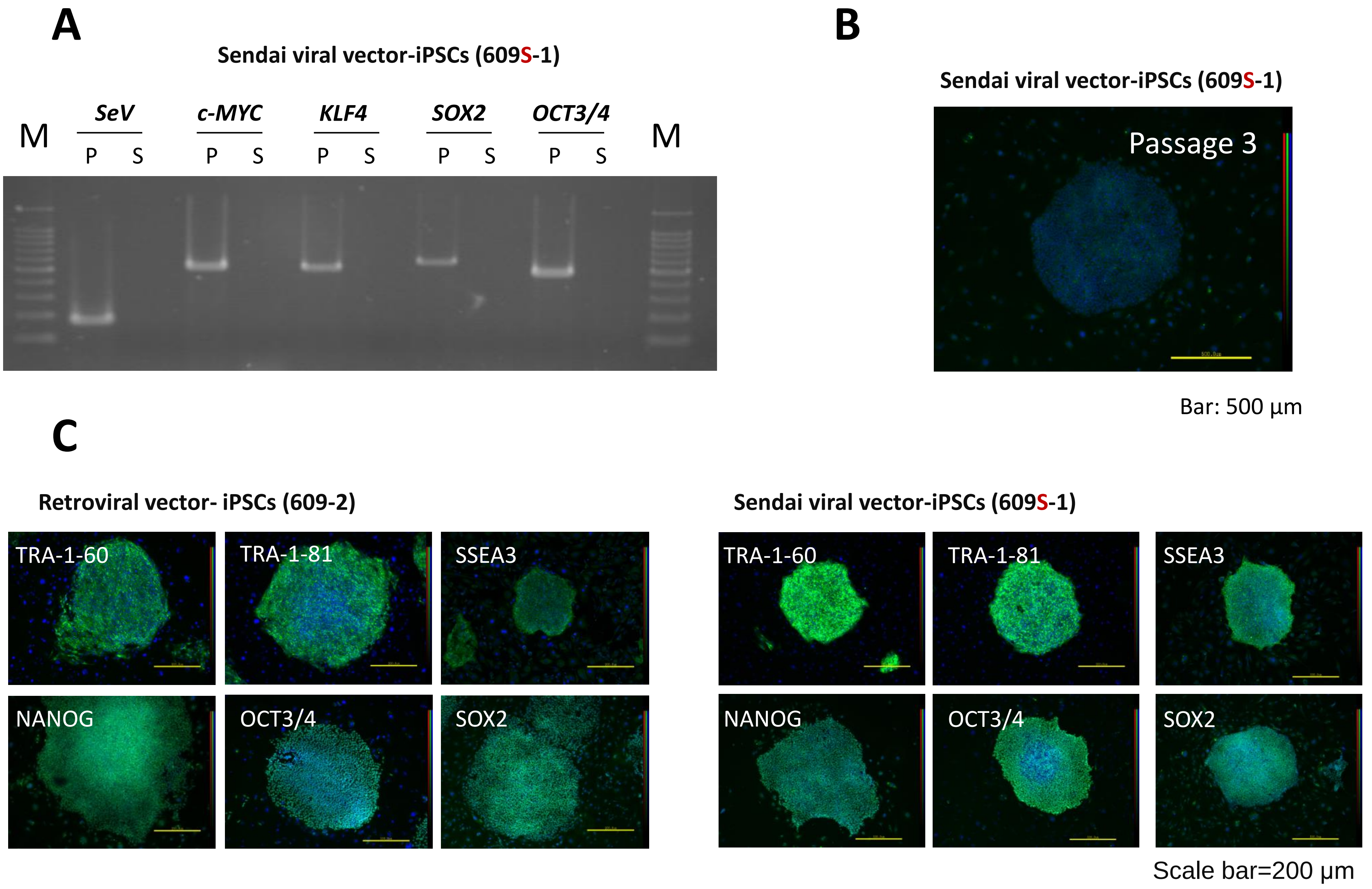

Supplementary Figure 3

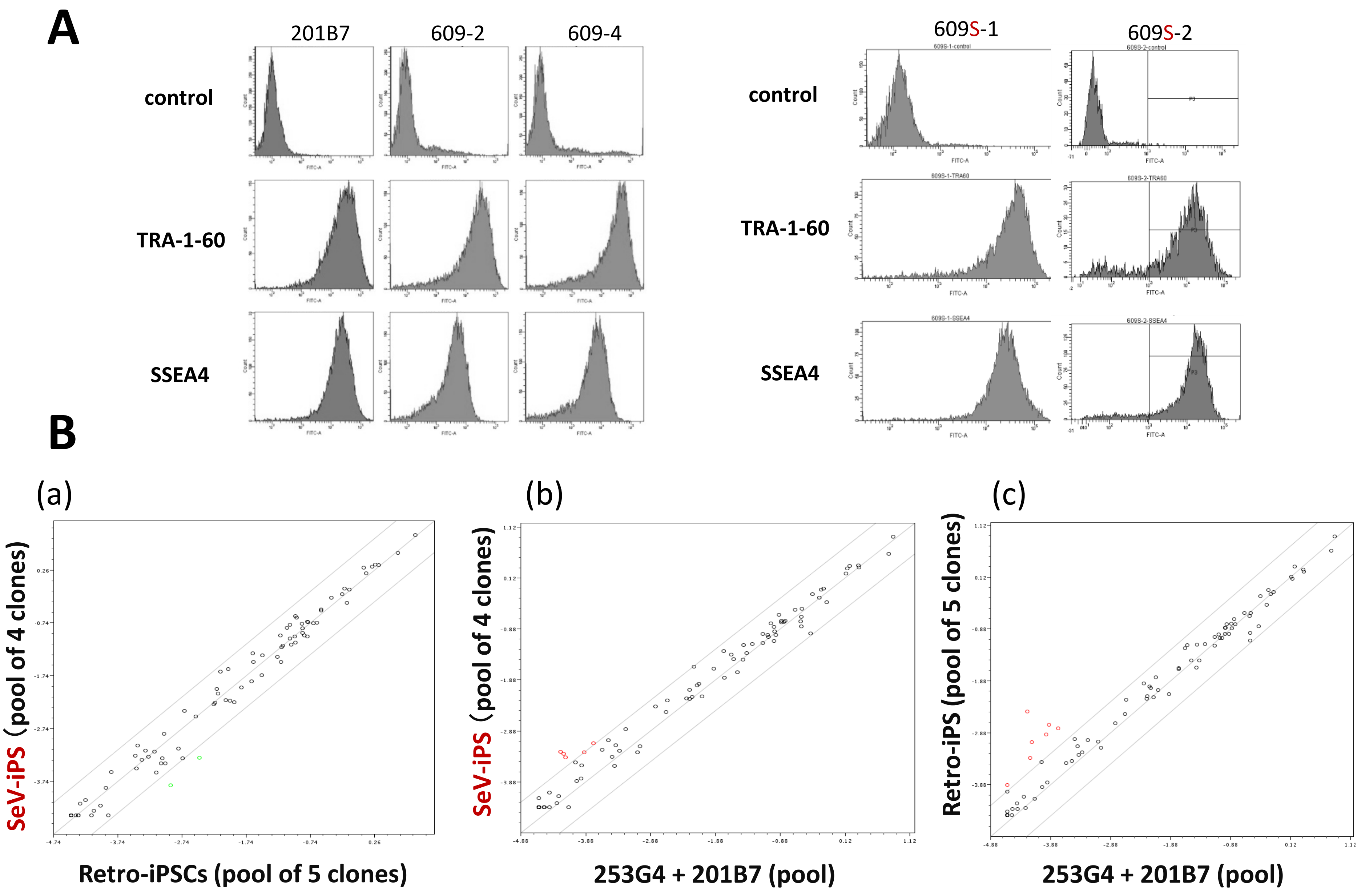

Supplementary Figure 4

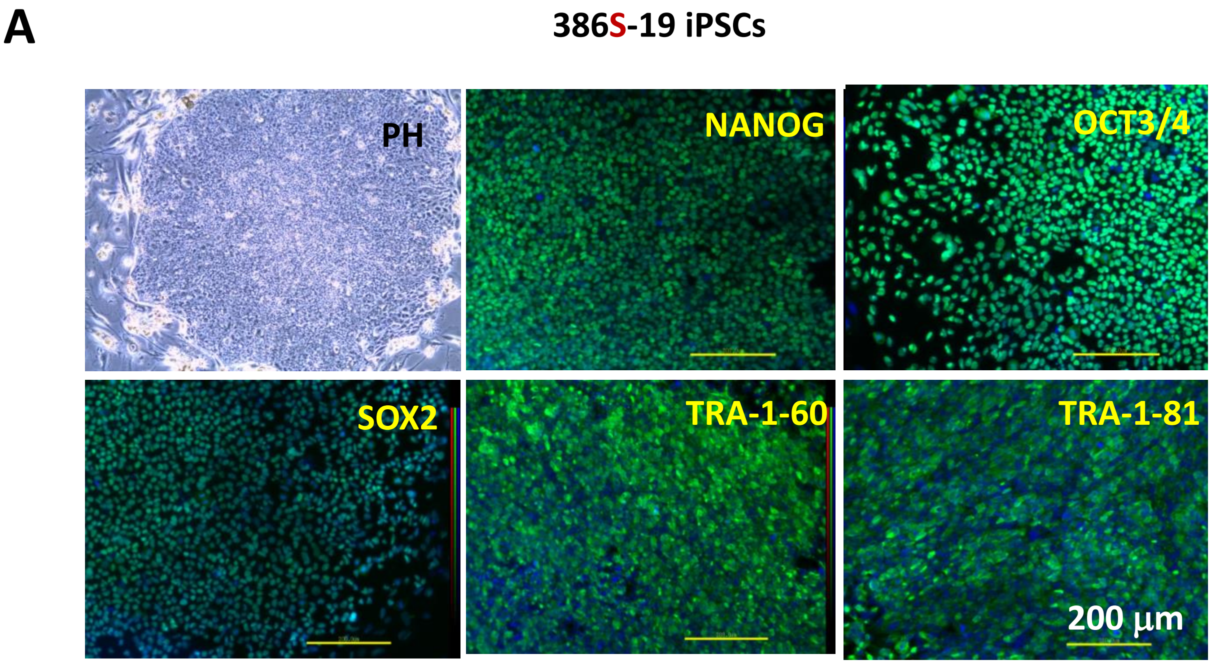

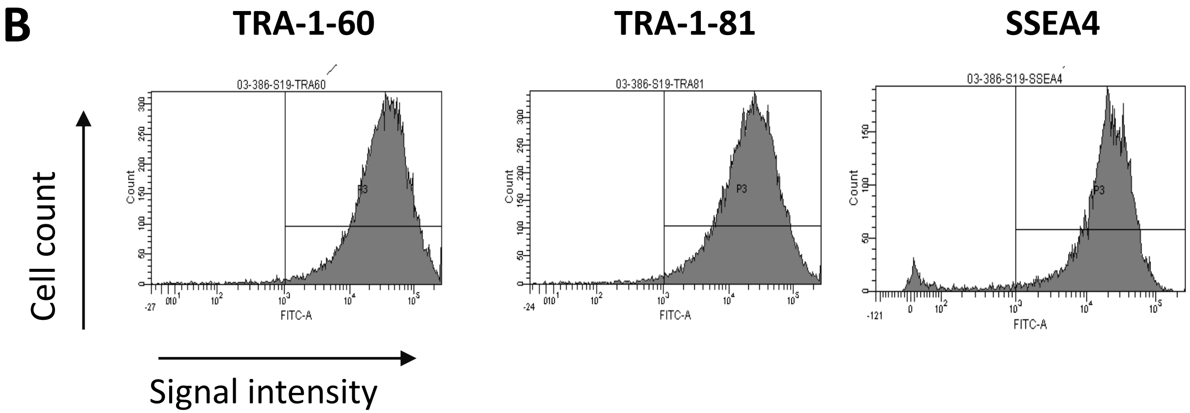

Supplementary Figure 5

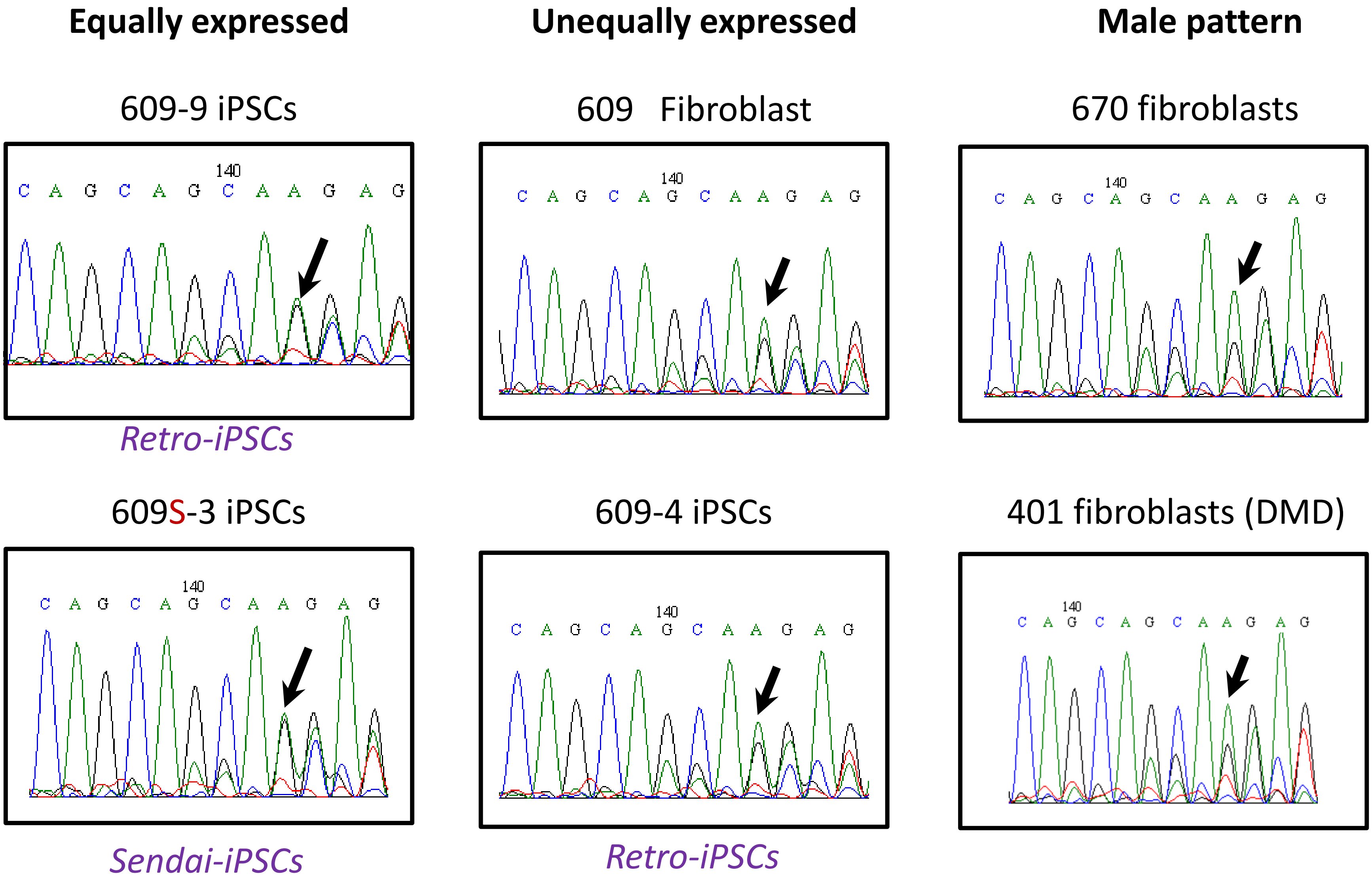

Supplementary Figure 6

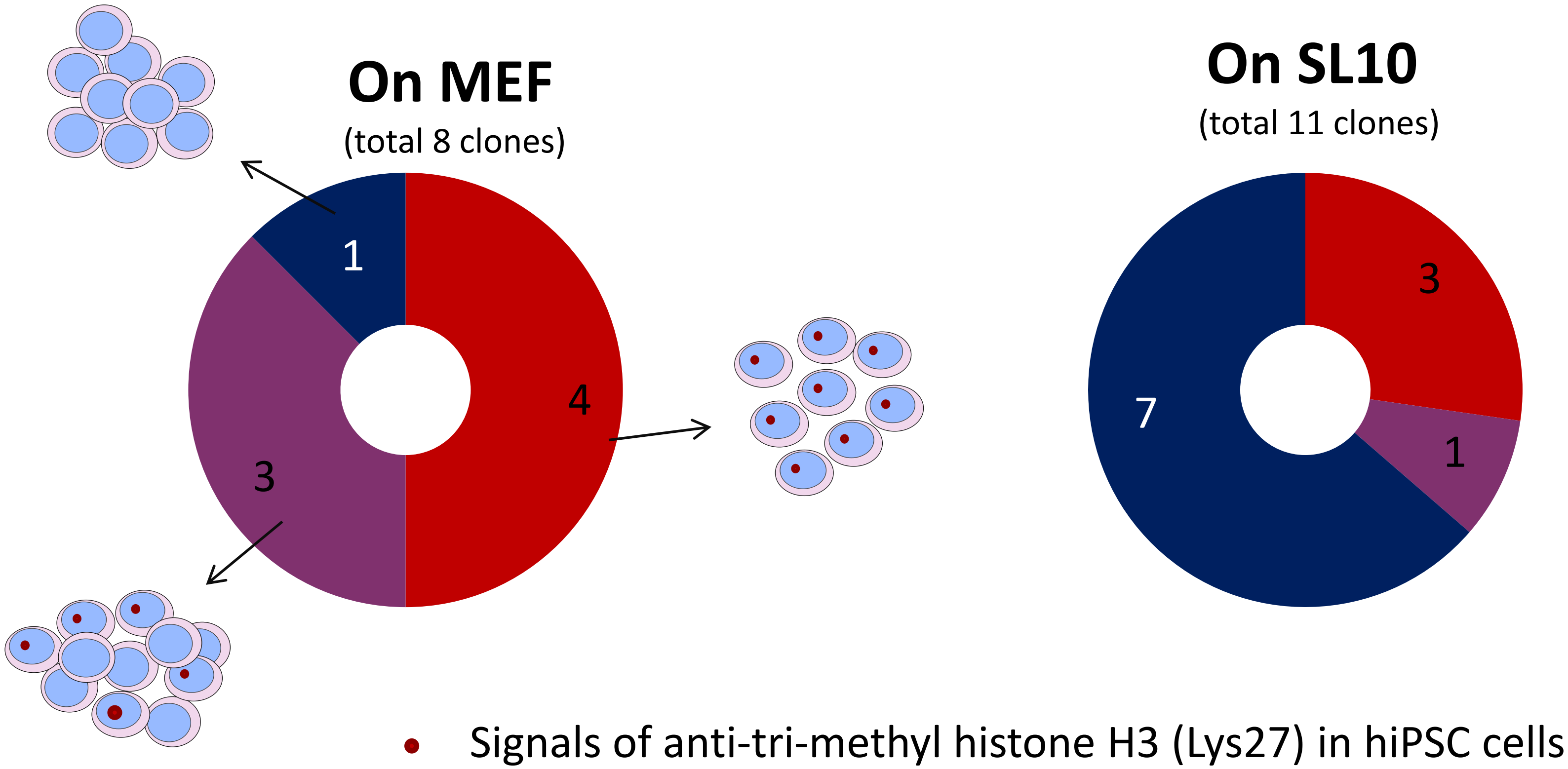

Supplementary Figure 7

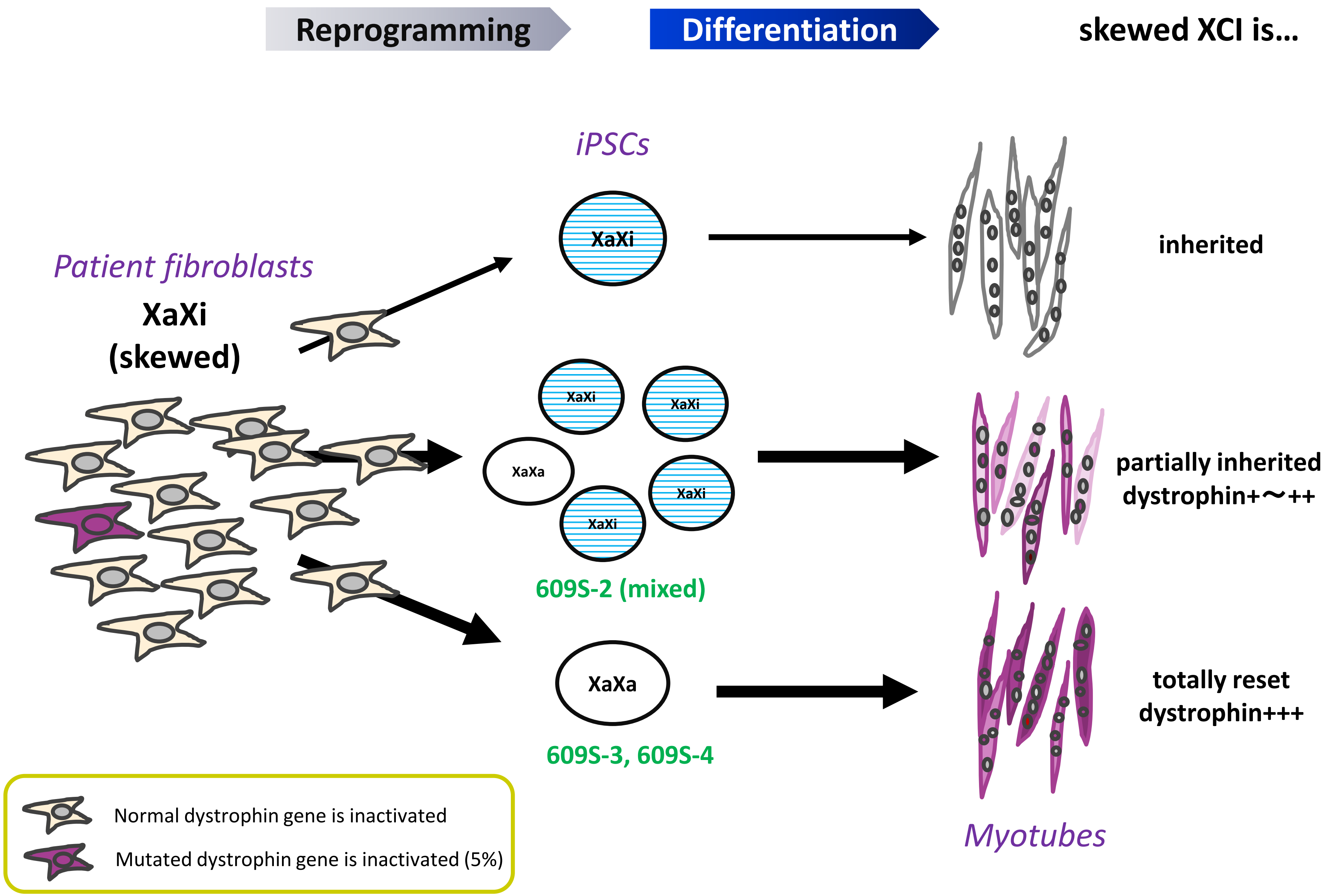

Supplement: Supplementary file 1 — Supplementary TABLE 1 X-chromosome signals in the nucleus of fibroblasts isolated from a DMD-manifesting carrier (609) and in four hiPS clones. [file 7906843.f1.pdf]
